# Systematic review of interventions to increase the use of arteriovenous fistulae and grafts in incident haemodialysis patients

**DOI:** 10.1177/11297298211006994

**Published:** 2021-04-12

**Authors:** Jonathan De Siqueira, Alexander Jones, Mohammed Waduud, Max Troxler, Deborah Stocken, David Julian A Scott

**Affiliations:** 1 Leeds Institute of Cardiovascular and Metabolic Medicine, University of Leeds, Leeds, UK; 2 Leeds Vascular Institute, Leeds Teaching Hospitals NHS Trust, Leeds, UK; 3 Department of Vascular Surgery, Bradford Teaching Hospitals NHS Foundation Trust, Bradford, UK; 4 Leeds Institute of Clinical Trials Research, University of Leeds, Leeds, UK

**Keywords:** AV fistula, catheters, dialysis, prosthetic grafts, economics and health services, nursing

## Abstract

**Background:**

Patients who commence haemodialysis (HD) through arteriovenous fistulae and grafts (AVF/G) have improved survival compared to those who do so by venous lines.

**Objectives:**

This systematic review aims to assimilate the evidence for any strategy which increases the proportion of HD patients starting dialysis through AVF/G.

**Data sources:**

Medline, Embase, Cochrane Central and Scopus.

**Study eligibility, participants and interventions:**

English language studies comparing any educational, clinical or service organisation intervention for adult patients with end stage renal failure and reporting incident AVF/G use.

**Study appraisal and synthesis:**

Two reviewers assessed studies for eligibility independently. Outcome data was extracted and reported as relative risk. Reporting was performed with reference to the PRISMA statement.

**Results:**

Of 1272 studies, 6 were eligible for inclusion. Studies varied in design and intervention. Formal meta-analysis was not appropriate. One randomised controlled trial and two cohort studies assessed the role of a renal access coordinator. Two cohort studies assessed the implementation of qualitive initiative programmes and one cohort study assessed a national, structured education programme. Results between studies were contradictory with some reporting improvements in incident AVF/G use and some no significant difference. Quality was generally low.

**Conclusions:**

It is not possible to reach firm conclusions nor make strategic recommendations. A comprehensive package of care which educates and identifies patients approaching dialysis in a timely manner may improve incident AVF/G use. An unbiased, robust comparison of different strategies for timing AVF/G referral is required.

## Introduction

Arteriovenous fistula (AVF) is considered the gold standard vascular access for haemodialysis (HD).^1^ AVF is associated with longer functional access, lower infection rates and reduced mortality.^2,3^ Patients initiating dialysis with permanent access in the UK have a 90-day mortality of 3.5%. By contrast, the mortality is doubled in patients starting with a tunnelled central venous catheters (CVC) (7%).^4^ Similar discrepancies are found in the USA, where 6-month incident HD mortality is 9% in AVF users and 32% in those using CVC, though it is argued that this may be partly due to differences in patient factors.^5^

Despite its benefits, arteriovenous fistulae and grafts (AVF/G) are associated with haemorrhage,^6^ congestive heart failure,^7^ steal phenomena^8^ and ischaemic neuropathy.^9^ Many fistulae require reintervention or fail to mature into functionally useful access altogether and the evidence for non-maturation risk factors is contradictory.^10–13^ Recent evidence suggests that the survival advantage of AV fistulae and grafts is lost in very frail or elderly patients^14^ and that there is substantial morbidity associated with using fistulae in these cohorts.^15^ There has therefore been a cultural shift in recent years towards an individualised approach to vascular access, rather than AVF for all.

In the USA, AVF formation is recommended in selected patients when estimated glomerular filtration rate (eGFR) reaches 15–20 mL/min/1.73 m^2^.^16^ Similar recommendations are made in Japanese Society guidelines,^17^ despite no high level evidence found on systematic review to support the use of laboratory markers as criteria for vascular access referral.^18^ By contrast, guidelines from the UK^19^ and continental Europe^20^ recommend that access should be formed 3–6 months before HD is expected to commence, that is based on a referring nephrologist’s clinical judgement, (including pre-empted difficulties in access formation), rather than laboratory thresholds alone. However, the high number of patients starting dialysis with CVC in the UK^21^ despite national audit standards to the contrary implies that clinical judgement alone is not an effective strategy for timing access. Other strategies to improve AV access uptake have been described, including focus on patient education, the implementation of quality initiatives^16^ and access co-ordinators,^22^ though the impact of these remains uncertain.

Given the complications associated with incident CVC use, contrasting international recommendations for AVF timing and consistently low number of patients starting dialysis with mature access, this systematic review aims to identify and assimilate the evidence for any intervention which increases the number of adult patients initiating HD with AVF/G.

## Methods

### Eligibility criteria

English-language randomised and non-randomised controlled trials, cohort studies, case-control studies and intervention-focused observational studies published at any time were eligible for inclusion. Studies must have assessed any relevant intervention (clinical, educational or service reorganisation) and reported the outcome of interest: the proportion of patients initiating HD via AVF/G. The comparator was ‘standard care’, or a similar descriptor. Studies were eligible regardless of publication status, though full texts with description of methodology and results were required for inclusion. Patients assessed must be aged older than 18, of any gender or ethnicity, with chronic kidney disease (CKD).

Studies were excluded if they reported outcomes for prevalent HD patients only. Studies describing interventions on paediatric patients could only be included if data for adult patients was available and could be examined separately. Single arm studies with no comparator were excluded.

### Identification of studies

The search strategy was developed with the assistance of an expert reference librarian. Electronic databases (Medline, Embase, Cochrane CENTRAL and Scopus) were searched through to June 2019 (Supplemental Appendix 1). Two reviewers (JDS, AJ) independently assessed abstracts for eligibility, according to a predefined protocol. Those abstracts thought to be eligible were retrieved in ‘full text’ form. Full texts were subsequently re-assessed, independently. Disagreements between reviewers were mediated through direct discussion. Where agreements could not be reached, a third author was available to arbitrate (DJAS, no arbitration necessary). Once consensus was reached, citations were manually forward- and back-searched for articles which met inclusion in the review.

### Data analysis and risk of bias

Design, intervention, comparator, setting, participant numbers, country of study and outcome data (proportion of incident haemodialysis patients starting HD with an AVF/G) were extracted from included studies in tabular form by a single reviewer (JDS). Where possible relative risks (RR) were calculated from the extracted data. No meta-analysis of reported summary data was carried out due to heterogeneity in interventions and poor descriptions of control arms. Thus, synthesis was performed in narrative form. Randomised controlled trials (RCTs) were assessed for bias using the Revised Cochrane Risk of Bias tool (RoB2).^23^ All other studies’ risk of bias was evaluated using the Newcastle-Ottawa Score.^24^ Tools were employed by reviewers (JDS, AJ) independently and agreed upon in the same manner as the eligibility of studies. Risk of bias across studies was synthesised narratively.

Reporting of the systematic review was carried out using the principles of the Preferred Reporting Items for Systematic Reviews and Meta-Analyses (PRISMA) statement (Supplemental Appendix 2).

## Results

### Study selection

**Figure 1.**
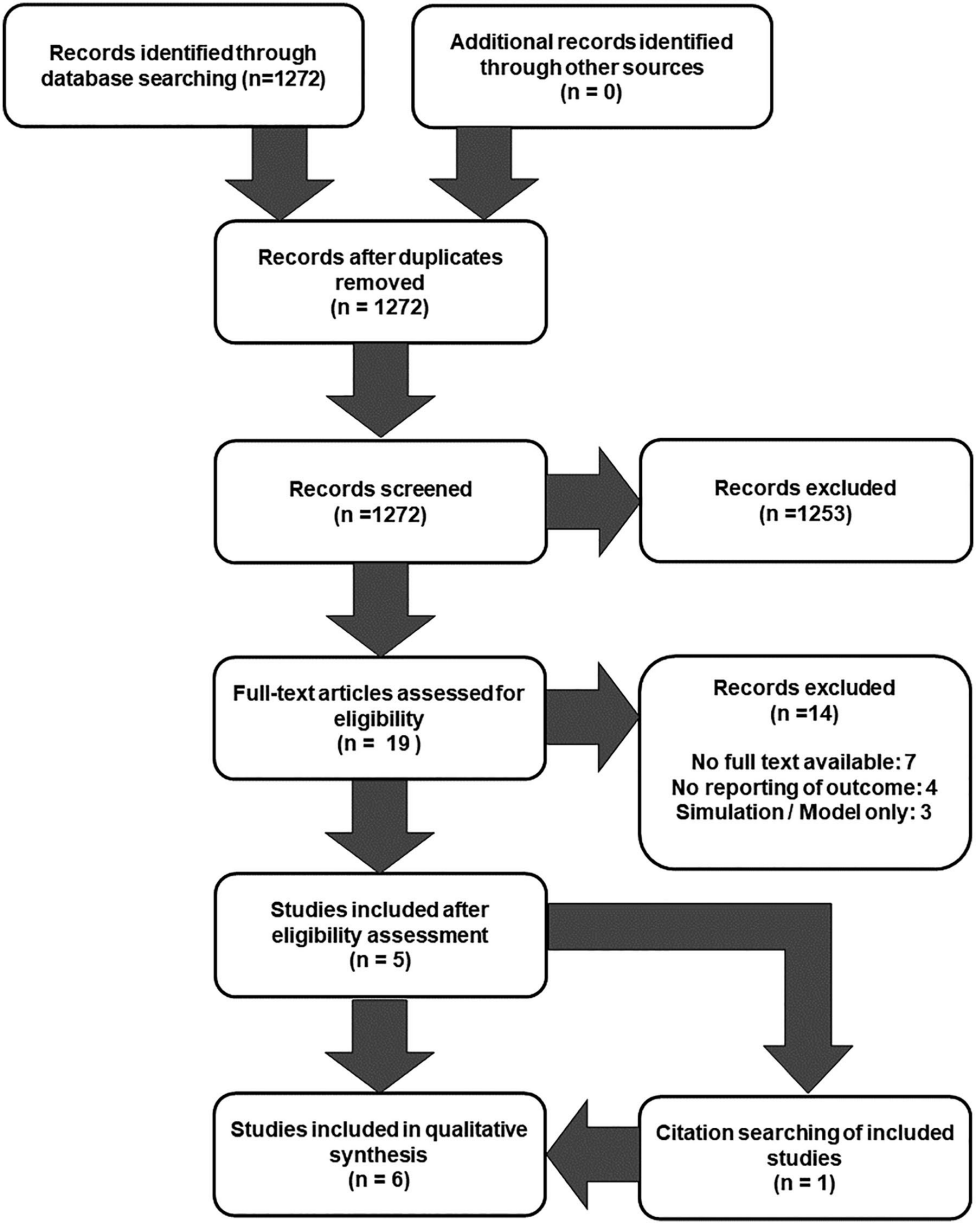
Algorithm for study identification.

The search strategy identified 1272 potential studies (Figure 1). After screening, discussion and conferment, 19 studies were selected for full text review, without the need for arbitration. Of these, five met eligibility criteria. Manual searching of citations led to one further study being identified (Figure 1, Table 1).The included studies consisted of one randomised controlled trial (RCT)^25^ and five cohort studies.^26–30^

**Table 1.** Included studies.

Author	Design	Intervention	Comparator	Setting	Participant numbers	Country	Outcome (exposure vs control)
Fishbane et al.^25^	Randomised Controlled Trial	Care co-ordinator	‘Usual care’	Patients approaching ESRF	130	USA	10/19 (53%) versus 7/26 (27%) incident AVF/G use (RR 1.95, 95% CI 0.91–4.19) (*p* = 0.09)
Gale et al.^26^	Retrospective cohort study	Care co-ordinator	Unmatched historical controls	Patients approaching ESRF	287	USA	19/44 (43%) versus 12/39 (31%) functional permanent access at initiation (RR 1.40, 95% CI 0.78–2.50) (*p* = 0.25)
Polkinghorne et al.^28^	Retrospective cohort study	Care co-ordinator	Unmatched historical controls	Patients approaching ESRF	184	Australia	63/84 (75%) versus 56/100 (56)% incident AVF use (RR 1.37, 95% CI 1.11–1.69) (*p* = 0.003)
Owen et al.^29^	Retrospective cohort study	Quality improvement programme	Unmatched historical controls	Patients approaching/with established ESRF	*Unclear*	Australia	83% versus 24% incident AVF use (*p* < 0.001)*
Ackad et al.^30^	Retrospective Cohort Study	KDOQI practice recommendations	Unmatched historical controls	Patients approaching/with established ESRF	134	USA	9/70 (12.9%) versus 3/64 (4.7%) incident AVF use (RR 2.54, 95% CI 0.72–9.01) (*p* *=* 0.14)
Lacson et al.^27^	Retrospective cohort study	Patient education programme	Matched contemporaneous controls	Patients approaching ESRF	5 600	USA	778/2800 (27.8%) versus 428/2800 (15.3%) incident AVF use (RR 1.78, 95% CI 1.60–1.97) (*p* < 0.0001)

ERF: end stage renal failure; AVF/G: arteriovenous fistula or graft; RR: relative risk; CI: confidence interval; KDOQI: kidney dialysis outcomes quality initiative.

* Taken from author’s stated calculation, not possible to verify result.

### Summary synthesis

One RCT (25) and two retrospective cohort studies (26, 28) assessed the role of care co-ordinators. Across the three studies, two (25, 26) failed to demonstrate a significant difference in the outcome of interest ((RR = 1.95, 95% CI = 0.91–4.19) (*p* = 0.09), (RR = 1.40, 95% CI = 0.78–2.50) (*p* = 0.25)) and one (28) demonstrated significant improvement (RR = 1.37, 95% CI = 1.11–1.69) (*p* = 0.003) (Table 1). The RCT was well conducted though underpowered for this review’s outcome of interest, the two cohort studies’ quality were judged low (26) or fair (28) (Table 2), due to lack of clarity regarding standard care and the comparability of cohorts. No firm conclusions could therefore be drawn regarding the impact of care co-ordinators on the outcome of interest.

**Table 2.** Newcastle-Ottawa scores of included cohort studies.

Study	Domain			Quality
	Selection	Comparability	Outcome	
Gale et al.^26^	★★★		★★	Poor
Lacson et al.^27^	★★	★	★★★	Fair
Polkinghorne et al.^28^	★★	★	★★★	Fair
Owen et al.^29^	★★		★	Poor
Ackad et al.^30^	★★		★★	Poor

Two cohort studies^29,30^ reported incident AVF/G use following the introduction of QI programmes; One of these^30^ assessed KDOQI practice guidelines the other,^29^ assessed a locally devised programme. Only one study presented data in such a way where it could be extracted; no significant changes were demonstrated (RR = 2.54, 95% CI = 0.72–9.01, *p* = 0.14).^29^ The second study reported percentages only and secondary analysis could not be performed. Quality was judged poor in both studies (Table 2), predominantly due to a lack of comparability between cohorts. Contradictory findings, low level of evidence and high risk of bias limit conclusions regarding the impact of QI programmes.

Lastly, one fair quality study^27^ demonstrated a significant improvement (RR = 1.78, 95% CI = 1.60–1.97, *p* < 0.0001) in permanent access as a result of a structured education programme, when compared to contemporaneous matched controls. Though a large number of patients were analysed, they appeared to originate from different geographical cohorts and control group education was not described fully.

Risk of bias across all included studies was judged to be high due to the risk of publication bias in intervention-focused historical cohort studies.

### Narrative

#### Care coordinators

A single, unblinded, parallel group, RCT^25^ assessed the role of a care co-ordinator in 130 patients approaching ESRF, in New York State. The care co-ordinator delivered one-to-one patient education sessions, dietary information, consolidated medication and monitored patient weight.^25^ Its primary outcome measure was hospitalisation rate, though secondary outcomes included initial vascular access at commencement of dialysis. Of 59 patients who reached ESRF, 45 started haemodialysis. There was a non-significant increase in incident AVF/G use at first dialysis in the intervention group (10/19 vs 7/26, RR = 1.95, 95% CI = 0.91–4.19, *p* = 0.09). This RCT was judged to have a low risk of bias: patients were appropriately randomised, there were few protocol violations and analysis was on intention to treat. All outcome data and adverse events were fully accounted for. The only notable flaw was a poor description of the control group’s ‘usual care’ and to what degree they may have received the same or similar interventions from other clinicians.

Two retrospective observational studies also compared outcomes after the introduction of a care coordinator. In the first, conducted in Palo Alto, CA,^26^ the co-ordinator delivered education, identified appropriate diagnostic testing and surgical review, provided motivation and post-operative follow-up. Some 131 patients receiving the intervention were compared to 156 historical controls matched by eGFR and predicted risk of ESRF. Of these, 19/44 and 12/39 of the intervention and control cohorts respectively, initiated dialysis with functioning, ‘permanent access’ (RR = 1.4, 95% CI = 0.78–2.50, *p* = 0.25). The quality of evidence was judged poor (Table 2): Follow up was insufficient to identify all patients progressing to haemodialysis and there was no description of how standard care differed from coordinator-lead care.

In the second study, conducted in Victoria, Australia, a care coordinator maintained a database of pre-ESRF patients, timed and co-ordinated referral for access, maintained the surgical waiting list and arranged follow up.^28^ 63/84 of the intervention cohort and 56/100 of the historical, unmatched control cohort met the outcome of interest (RR = 1.37, 95% CI = 1.11–1.69, *p* = 0.003). After adjustment for age, gender, late referral, aetiology of ESRF and type of presentation, a greater proportion of patients received dialysis with an AVF following the introduction of a care co-ordinator (OR = 2.85 95% CI = 1.32–6.15, *p* = 0.008). This study was judged to present fair quality evidence (Table 2). Though there was controlling for baseline characteristics, there was no indication of whether interventions overlapped.

#### QI programmes

Two cohort studies investigated the impact of a QI programme. Both programmes emphasised referral to nephrology services at CKD stage 4, early patient education and early referral for vascular access (e.g. eGFR < 25 mL/min/1.73 m^2^). In the first,^28^ conducted in New Jersey, USA, 70 patients commencing dialysis after the establishment of KDOQI were compared to 64 historical controls. The use of AV Fistulae at first dialysis increased (3/64 vs 9/70, RR = 2.54 95% CI = 0.72–9.01), though not significantly (*p* = 0.14). Study quality was poor (Table 2), due to the use of historical controls, the absence of matching or adjustment for baseline factors or completeness of follow up. Standard practice prior to the implementation of practise guidelines was incompletely described.

The second QI study took place in Victoria, Australia.^30^ 595 patients commencing dialysis before, during and after the implementation of a QI programme were compared over a 4-year period. The proportion of patients starting dialysis with an AV fistula increased from 24% to 83%. No absolute numbers are given within the manuscript. Study quality was poor (Table 2). There was no description of differences in cohorts, matching or adjustment and it was not possible to judge completeness of follow up. There was a lack of clarity regarding standard practice prior to the introduction of the QI programme.

#### Standardised education

One large cohort study assessed the impact of a national standardised education programme for pre-ESRF patients^27^ in the USA. About 2800 matched pairs were selected from a pool of 32,617 patients starting dialysis over a 16-month period. The intervention group was provided with four education sessions regarding ESRF treatment choices over a 180-day period by trained educators. Contemporaneous controls commenced HD via a standard pathway which did not include the national educational programme. There was a significant difference in the number of HD starters who did so via AVF (778/2800 vs 428/2800, RR = 1.82 95% CI = 1.60–1.97; (*p* < 0.0001)). This study also reported improved 90-day survival in the intervention group (Hazard ratio 0.61, 95% CI = 0.5–0.74). Overall quality was judged to be fair (Table 2): This was a well performed study with robust patient matching and outcome reporting. Nonetheless, the description of the control cohort’s education was vague and patients appeared to be drawn from different healthcare institutions in control and intervention cohorts.

## Discussion

National practice guidelines recommend that suitable patients should commence HD with functional AVF:^16,17,19,20^ This systematic review aimed to identify any evidence-based strategy to facilitate this recommendation. Given the multifaceted and overlapping nature of the interventions, the absence of a consistent outcomes across studies and low level and quality of evidence, it has not been possible to identify any single action or criteria which achieves this.

Of the six studies identified, three cohort studies^28–30^ used renal function threshold as a criteria for vascular access referral, as part of a broader package of care. This strategy is recommended by American and Japanese Guidelines.^16,17^ In two studies,^28,29^ there was a significant increase in AVF use. However, it is not possible to distinguish what impact this single intervention had, given coexisting interventions or how this should influence future policy.

Lacson et al.^27^ highlighted that patients undergoing structured pre-ESRF education had a notably higher survival (HR = 0.6, 95% CI = 0.50–0.74, in favour of the intervention group). Analysis was based on matched, contemporaneous groups. The impact of vascular access on patient survival is currently subject to debate^16^ though the preserved advantage in these matched cohorts implies it is not discountable.

A limitation common to all included studies was the poor description of ‘standard care’. Without such information, it is not possible to discern whether practice and outcomes changed significantly as a result of the interventions described, or because of external factors such as observation bias. Further, the lack of clarity over control groups, impeded pooling of studies for analysis. The included studies were also notable for the absence of adverse event reporting. Lastly, not all outcome data could be extracted and synthesised, due to one included study only reporting secondary results, as percentages. Combined with the heterogeneity of studies, this has limited the synthesis of data.

## Conclusions and research recommendations

There is low-to-fair-quality, low-level evidence to suggest that a broad package of care including patient education, firm criteria regarding the timing of vascular access referral and surgical waiting list optimisation may increase the number of patients starting HD with permanent access, though firm conclusions cannot be drawn. The question as to when best to refer suitable patients for vascular access formation remains unanswered. The studies highlighted demonstrate a consistently low level of incident AVF use, suggesting that current timing strategies are inadequate.

There remains a need to develop a robust, validated model for predicting when patients are likely to require dialysis. A randomised controlled trial assessing clinical judgement (UK/European guidelines) against eGFR threshold (USA/Japanese guidelines) in the timing of vascular access referral is needed to support decision making in pre-dialysis care, develop a prediction model and lend evidence to current practice guidelines.

## Supplemental Material

sj-docx-1-jva-10.1177_11297298211006994 – Supplemental material for Systematic review of interventions to increase the use of arteriovenous fistulae and grafts in incident haemodialysis patients Click here for additional data file. Supplemental material, sj-docx-1-jva-10.1177_11297298211006994 for Systematic review of interventions to increase the use of arteriovenous fistulae and grafts in incident haemodialysis patients by Jonathan De Siqueira, Alexander Jones, Mohammed Waduud, Max Troxler, Deborah Stocken and David Julian A Scott in The Journal of Vascular Access
