# Supplementary material for: Systematic review of interventions to increase the use of arteriovenous fistulae and grafts in incident haemodialysis patients
Source: J Vasc Access. 2021 Apr 12;23(5):832–8. doi: 10.1177/11297298211006994 (PMC9465552; doi:10.1177/11297298211006994)
Supplement: sj-docx-1-jva-10.1177_11297298211006994 – Supplemental material for Systematic review of interventions to increase the use of arteriovenous fistulae and grafts in incident haemodialysis patients [file sj-docx-1-jva-10.1177_11297298211006994.docx]

Appendix: Search Strategy

**EMBASE, Medline, CENTRAL**

| 1 | Arteriovenous Shunt, Surgical/ |
| --- | --- |
| 2 | Arteriovenous Fistula/ |
| 3 | Blood Vessel Prosthesis/ |
| 4 | (fistula* or AVF* or graft or grafts or shunt or shunts).tw |
| 5 | (vascular access or venous access).tw. |
| 6 | (dialysis access or hemodialysis access or haemodialysis access).tw. |
| 7 | or/1-6 |
| 8 | Renal Dialysis/ |
| 9 | exp Hemofiltration/ |
| 10 | dialysis.tw |
| 11 | predialysis.tw. |
| 12 | (hemodialysis or haemodialysis).tw. |
| 13 | (hemofiltration or haemofiltration).tw. |
| 14 | (hemodiafiltration or haemodiafiltration).tw. |
| 15 | Renal Replacement Therapy/ |
| 16 | peritoneal dialysis/ |
| 17 | or/8-16 |
| 18 | "Referral and Consultation"/ |
| 19 | (refer or referral* or referred).tw. |
| 20 | consult*.tw. |
| 21 | Time-to-Treatment/ |
| 22 | (time adj2 treat*).tw |
| 23 | or/18-22 [referral terms] |
| 24 | 7 and 17 and 23 |
| 25 | randomized controlled trial.pt |
| 26 | controlled clinical trial.pt. |
| 27 | randomized.ab. |
| 28 | randomly.ab. |
| 29 | trial.ab. |
| 30 | groups.ab |
| 31 | Comparative studies/ |
| 32 | Follow-up studies/ |
| 33 | Time factors/ |
| 34 | (preoperat* or pre operat*).mp. |
| 35 | chang*.tw |
| 36 | evaluat*.tw. |
| 37 | reviewed.tw. |
| 38 | prospective*.tw. |
| 39 | retrospective*.tw. |
| 40 | baseline.tw. |
| 41 | cohort.tw. |
| 42 | case series.tw. |
| 43 | 25 or 26 or 27 or 28 or 29 or 30 or 31 or 32 or 33 or 34 or 35 or 36 or 37 or 38 or 39 or 40 or 41 or 42 |
| 44 | 24 and 43 |
| 45 | remove duplicates from 44 |

**Scopus:**

("Arteriovenous Shunt, Surgical"  OR  "Arteriovenous Fistula"  OR  "Blood Vessel Prosthesis”)  AND  ("Renal Dialysis"  OR  "Hemofiltration"  OR  "Renal Replacement Therapy"  OR  "Peritoneal Dialysis")  AND  ("Referral and consultation"  OR  "time to treatment")  AND  ("Randomized Controlled Trial"  OR  "comparative studies"  OR  "Follow-up Studies"  OR  "time factors")
